# Azathioprine and hydroxychloroquine overdose in Sjögren’s syndrome patient with hypocalcemia: a case report

**DOI:** 10.1186/s13256-024-04390-w

**Published:** 2024-02-27

**Authors:** Alireza Kooshki, Omid Mehrpour, Samaneh Nakhaee

**Affiliations:** 1 Student Research Committee, Birjand University of Medical Sciences, Birjand, Iran; 2 Michigan Poison & Drug Information Center, School of Medicine, Wayne State University, Detroit, MI USA; 3 Medical Toxicology and Drug Abuse Research Center (MTDRC), Birjand University of Medical Sciences, Birjand, Iran

**Keywords:** Hydroxychloroquine, Azathioprine, Overdose, Sjögren’s syndrome

## Abstract

**Introduction:**

Hydroxychloroquine and azathioprine have been routinely used to control and treat primary and secondary Sjögren’s syndrome, which potentially triggered some overdoses by these drugs. Toxicity from hydroxychloroquine and azathioprine manifests in the form of cardiac conduction abnormalities, nausea, vomiting, and muscle weakness. Recognizing these unique drug overdoses and management of these toxicities is important. This case report aims to expand our current understanding of these drug overdoses and their management and also underscores the importance of anticipating and identifying fewer common complications, such as hypocalcemia.

**Case report:**

A 34-year-old Persian woman with a history of Sjögren’s syndrome presented to the emergency department 3.5–4 hours after an intentional overdose of hydroxychloroquine and azathioprine and severe hypotension and loss of consciousness. Although the patient was regularly taking other medications, such as fluoxetine, naproxen, and prednisolone, she explicitly clarified that these were not the substances involved in her overdose. Early investigations showed hypokalemia (2.4 mEq/L), hypocalcemia (7.5 mg/dL), and hypoglycemia (65 mg/dL). She was also diagnosed with metabolic acidosis and respiratory alkalosis. The electrocardiogram showed changes in favor of hypokalemia; other lab tests were run on the patient. Supportive treatments were applied, including rapid intravenous fluid dextrose 5%, normal saline, potassium chloride 30 mEq, and calcium gluconate 100 mg. The patient was managed and monitored overnight in the emergency room and recovered without residual side effects.

**Conclusion:**

Hydroxychloroquine and azathioprine toxicity are considered rare, but it is likely to increase in frequency given the prevalence and increase in autoimmune diseases and the increasing usage of these drugs in treating such diseases. We found hypocalcemia as the presentation to this patient, which needs further investigation into the probable mechanism. Clinicians need to consider the unique effects of hydroxychloroquine and azathioprine poisoning and initiate appropriate emergency interventions to improve the outcomes in similar patients.

## Introduction

Sjögren's syndrome is a prevalent autoimmune disease characterized by dry eyes and mouth, often requiring treatment with disease-modifying antirheumatic drugs (DMARDs), such as hydroxychloroquine (HCQ) and azathioprine [1]. Misuse or overdose of these therapeutic agents can precipitate serious complications, including hypocalcemia, a condition rarely associated with Sjögren’s syndrome in the existing medical literature [2]. This case report discusses an exceptional case of HCQ and azathioprine overdose in a patient with Sjögren’s syndrome, aiming to provide crucial insights into managing such overdoses and their potential complications.

HCQ, initially an antimalarial drug, is now used in managing rheumatic diseases by modifying the pH of acidic vesicles and inhibiting receptor-mediated endocytosis, which in turn affects Toll-like receptor signaling (TLR) and cytokine production [3–6]. While HCQ overdose typically results in hypokalemia, hypoglycemia, and acidosis [2, 7], this case study presents an unusual instance of hypocalcemia. As the therapeutic window for HCQ is low, this drug is responsible for 10–30% of deaths in cases of overdose, and it is mostly due to the cardiac effect and electrolyte imbalance [8]. On the other hand, azathioprine curbs white blood cell proliferation by inhibiting purine synthesis and has a broad range of applications, from transplantation to rheumatic diseases [9]. Overdoses with azathioprine often manifest as nausea, vomiting, and mild leukopenia [10, 11], contributing to the complexity of this case. There are little-to-no drug interactions reported for these drugs, which makes them a commonly prescribed medication in autoimmune diseases [12].

Herein, we present a case of a patient with an intentional overdose of HCQ and azathioprine with low blood pressure in a patient with Sjögren’s syndrome. She was brought to the emergency room by paramedics and accompanied by a family member, presenting with severe hypotension and lethargy. Although the toxicology urine test was reported positive in some tests based on the previous studies and the patient’s statement that the overdose was just by HCQ and azathioprine, we considered these two drugs to be mainly responsible for the overdose. This case report aims to expand our current understanding of these drug overdoses and their management, and it also underscores the importance of anticipating and identifying fewer common complications, such as hypocalcemia.

## Case report

A 34-year-old Persian woman with a history of depression and Sjögren’s syndrome, currently being treated with fluoxetine, was brought to the emergency department (ED) by paramedics, presenting with nausea, hypotension, confusion, and drowsiness. Diagnosed with depression by a psychologist 6 months earlier, she had no previous history of overdose or self-harm. Approximately 4 hours before she arrived at the ED, she had taken about 40 hydroxychloroquine (HCQ) tablets (200 mg each) and 60 azathioprine tablets (50 mg each). Her baseline electrolyte levels 80 days before the incident were normal, indicating no prior hypocalcemia or hypokalemia.

Additionally, there were no previous instances of overdose, self-harm, or cardiac complications, further suggesting that the overdose was the cause of her current condition. She had vomited twice since the overdose. She had been under prednisolone, nonsteroidal anti-inflammatory drugs (NSAIDs, such as naproxen), and other painkillers for her pain management, but she stated that she did not overdose on these drugs. She had a family history of rheumatoid arthritis, but there was no family history of mental disorders.

Upon arrival, her vital signs were as follows: a heart rate of 62 beats per minute, blood pressure of 70/50 mmHg, respiratory rate of 19 breaths per minute, and oxygen saturation of 97%. Her body temperature was 36.7 °C, and she weighed approximately 58 kg. Physical examination showed mydriasis with reactive pupils, a weak but normal-sounding heart, and weak pulses in both hands. She complained of lip and finger stiffness, with a positive Trousseau sign and a negative Chvostek’s sign observed. Initial electrocardiography (ECG) showed a mildly flattened T wave and prolonged QT interval (640 milliseconds). The echocardiography showed an ejection fraction (EF) of 55% and pulmonary arterial pressure (PAP) of 25 mmHg. No clonus, tremors, or signs of hyperreflexia were observed. Neurological examinations revealed weakness in her extremities and a Glasgow Coma Scale (GCS) score of 13, indicating she was confused but responsive to voices.

Laboratory analyses of her blood and urine (Table 1) showed hypokalemia (potassium: 2.4 mmol/L), hypocalcemia (calcium: 7.5 mg/dL), hypoglycemia (blood glucose: 65 mg/dL), and a positive result for benzodiazepine, tramadol, buprenorphine, and morphine. She stated that she had not taken any of these substances and overdosed on azathioprine and HCQ only. Her blood gas analysis indicated metabolic acidosis with respiratory alkalosis. Complete blood count (CBC) revealed low levels of hemoglobin, red blood cells (RBC), hematocrit (HCT), total iron-binding capacity (TIBC), and iron levels. We also managed to find the patient’s last routine laboratory tests (80 days prior), in which all her findings were normal and indicated no hypocalcemia or hypokalemia before the incident. However, since there were no cardiac complications in the patient before the overdose, there was no previous ECG. She was transferred to the hospital’s poison control center owing to her initial findings (Table 1). Our data, as depicted in Table 1, indicate that the patient’s baseline electrolyte levels, including calcium (9.6 mg/dL) and potassium (3.5 mmol/L), were within normal limits 80 days prior to the incident. She received intravenous ondansetron (8 mg), pantoprazole (40 mg), potassium chloride injection (30 mEq), and a mixture of 5% dextrose and 0.9% sodium chloride. Her blood pressure improved to an acceptable level overnight following the administration of 1000 cc each of 5% dextrose and 0.9% sodium chloride. An infusion of 100 mg of calcium gluconate, diluted in 5% dextrose, ameliorated her finger stiffness. Post potassium chloride injection, her QT interval shortened to 590 milliseconds, and the flattening of T waves improved.

**Table 1:** Laboratory values during hospitalization and follow-up of the patient

Laboratory parameters	Reference values	80 days before the incident	Hospital day 1 (20:30)	Hospital day 2 (6 am)	Hospital day 2 (6 pm)	Seven days later, a follow-up
Glucose (mg/dL)	70–135	87	65	–	81	85
Urea (mg/dL)	15–45	15	21	33.0	13	26
Creatinine (mg/dL)	0.6–1.3	0.60	0.90	0.74	0.72	0.61
Sodium (mEq/L)	135–145	–	142	145	140	142
Potassium (mEq/L)	3.5–5.0	3.5	2.4	4.2	3.8	3.9
Calcium (mg/dL)	8.5–10.2	9.6	7.5	–	9.3	9.5
Magnesium (mg/dL)	1.7–2.2	–	–	–	2.4	–
Phosphorus (mg/dL)	2.8–4.5	–	–	–	2.5	–
Troponin	Qualitative	–	Negative	Negative	Negative	Negative
CRP	Qualitative	1.23	Negative	Negative	Positive (+ 1)	Negative
Fe (mcg/dL)	60–170	–	–	16	–	–
TIBC (mcg/dL)	240–450	–	–	204.0	–	–
ALP	44–147 IU/L	46	–	71	–	45
ALT (IU/L)	< 31	14.6	–	16	–	18.2
AST (IU/L)	< 31	20.9	–	40	–	19.4
Venous blood gas (VBG)						
PH (mmHg)	7.31–7.41	–	7.430	7.350	–	–
pCO_2_ (mmHg)	40–52	–	21.2	30.7	–	–
pO_2_ (mmHg)	30–50	–	146.6	96.0	–	–
HCO_3_ (mEq/L)	22–26	–	14.1	16.9	–	–
BE (mEq/L)	−4 to +4	–	−7.9	−7.3	–	–
O_2_ saturation (%)	95–100	–	98.9	96.8	–	–
TCO_2_ (mEq/L)	24.3–30.5	–	14.7	17.8	–	–
SBE (mEq/L)	−2 to +2	–	−8.6	−7.4	–	–
BB (mmol/L)	48–49	–	38.1	38.3	–	–
Coagulation						
PTT (seconds)	23–42	–	30	–	30	–
PT (seconds)	11–14.7	–	12.9	–	12.0	–
INR	–	–	0.99	–	1.0	–
Hematology						
ESR (mm/hour)	< 20	23	–	–	20.0	–
WBC	4.5–11	4.40	12.4	9.14	5.8	5.3
RBC	4.0–5.9	4.15	3.62	3.23	3.43	4.13
HGB	12.0–17.5	12.4	10.2	9.8	10.6	12.5
HCT	36.0–50.0	36.6	30.6	28.0	30.3	37.0
MCV	80.95	88.2	84.5	86.7	88.3	89.6
MCH	27.0–33.0	29.9	28.2	30.3	30.9	30.3
MCHC	33.0–36.0	33.9	33.3	35.0	35.0	33.8
PLT	150–450	222	175	165	175	255

CRP, C-reactive protein; TIBC, total iron-binding capacity; ALP, alkaline phosphatase; ALT, alanine aminotransferase; AST, aspartate transaminase; SBE, standard base excess; BB, buffer base; PT, prothrombin time; PTT, partial thromboplastin time; INR, international normalized ratio; ESR, erythrocyte sedimentation rate; WBC, white blood count; RBC, red blood cells; HGB, hemoglobin; HCT, hematocrit test; MCV, mean corpuscular volume, MCH, mean corpuscular hemoglobin, MCHC, mean corpuscular hemoglobin concentration; PLT, platelet

**Fig. 1:**
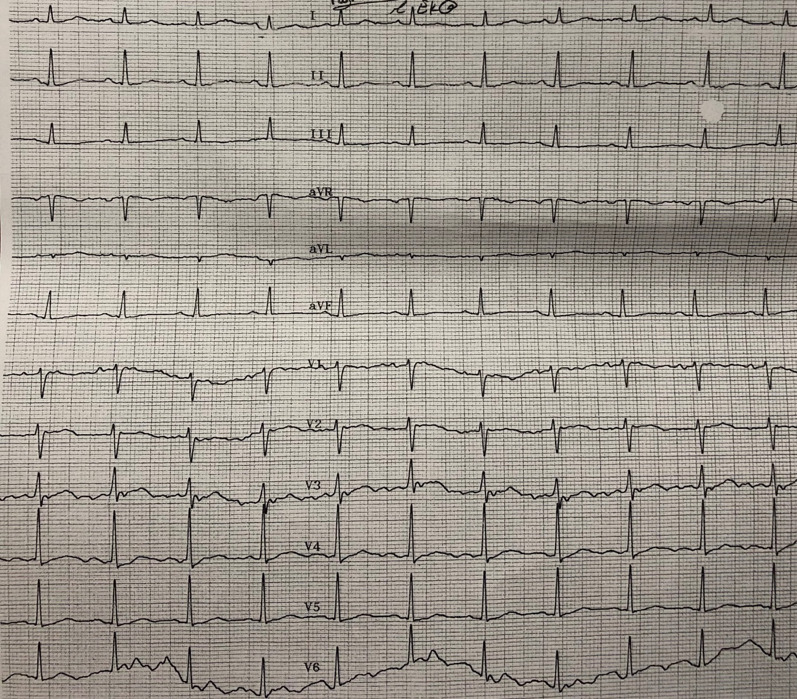
Patient’s electrocardiogram 24 hours after overdose

Approximately 24 hours after consuming HCQ and azathioprine, the patient reported nausea and headaches. She underwent a brain computed tomography (CT) scan and an ECG, which showed normal results (Fig. 1). She received injections of ketorolac (30 mg), dexamethasone (8 mg), and ondansetron (2 mL) for her symptoms (nausea and headache). About 26 hours after admission, the patient chose to be discharged. She was prescribed calcium, iron, and folate supplements and was subsequently referred to therapy sessions with her psychologist.

One week later, during follow-up, the patient had fully recovered, and her blood tests showed no significant abnormalities. Consent for the publication of this case has been obtained from the patient. We confirm that all experiments were carried out following relevant guidelines and regulations. Informed consent for participation and publication was obtained from the patient. Postrecovery, the patient’s treatment regimen for Sjögren’s syndrome was thoroughly reviewed and adjusted to minimize future risks, highlighting the importance of individualized management in complex cases. The absence of immediate preoverdose health data presents a limitation in our case, possibly obscuring a complete understanding of the patient’s baseline health status before the overdose.

## Discussion

The implications of this case go beyond merely an anomaly, opening up an avenue for further investigation into the intricate interplay between autoimmune diseases, their medications, and electrolyte imbalance. The enhanced understanding of this relationship will equip clinicians to manage similar cases better and possibly even contribute to optimizing treatment strategies. This case highlights the potential complexities of drug interactions in polypharmacy, particularly in autoimmune diseases, underscoring the need for vigilant monitoring and patient education about medication risks. Given the rarity of overdoses with these medications and the significant complications of HCQ and azathioprine on human health, this report can significantly aid physicians in this field. It can also prepare emergency doctors for similar encounters in the future.

HCQ is commonly used in controlling various autoimmune diseases, and poisoning with this drug may lead to hypokalemia and QT prolongation [13]. This drug has been used to treat malaria since World War II. It has recently gained attention because of its effect on connective tissue diseases and its antithrombotic and antineoplastic effects [14, 15]. However, it gained notable attention during the recent coronavirus disease 2019 (COVID-19) pandemic, as HCQ was more frequently used to treat and control the disease’s progression. HCQ has a wide spectrum of reversible and irreversible effects on the body depending on dosage and total duration [16]. This fact makes HCQ plausible for frequent intentional and unintentional overdoses. HCQ has metabolic effects like hypoglycemia, but the mechanism causing it is poorly known [7]. It has been suggested that an increase in C-peptide secretion and a decrease in insulin clearance and degradation rate can cause this effect [17]. Most cases of hypoglycemia linked to HCQ have been reported in patients with prediabetes or diabetes [18], although there were some cases in patients without diabetes [7]. These serious effects can be fatal for the patient if not managed properly. Consequently, the significance of this topic cannot be overlooked.

The patient presented with hypocalcemia and hypokalemia, which can justify the longer QT interval in her ECG. Several HCQ overdose cases reported hypokalemia [2, 13], but hypocalcemia has not been reported often [19]. Although the mechanism that caused hypocalcemia in this case is unclear, there are limited studies on HCQ and its relation with calcium. A study reported that HCQ could inhibit calcium-dependent signals in B and T cells by reducing store capacity and refiling [20]. Moreover, azathioprine also did not have a significant study effect on blood calcium balance. It is also known that alkalosis can promote calcium to bind to proteins, such as albumin, and reduce ionized calcium, but that does not change the total calcium blood level [21].

Azathioprine is converted in the body to 6-mercaptopurine and 6-thioinosinic acid, which inhibits DNA synthesis and, therefore, its affects mostly proliferating cells, such as white blood cells (WBC) and red blood cells (RBC) [10]. On the other hand, azathioprine overdose cases have been rare, but massive overdoses with this drug reported no significant complications aside from nausea, vomiting, and a decrease in WBC [22]. However, in this case, the WBC was slightly more than normal owing to the patient’s history of chronic autoimmune disease. Ultimately, overdose by azathioprine is very rare, and there are not enough studies to justify the outcome of this patient’s symptoms; therefore, further studies are needed to be settled on this subject.

While some outcomes in this patient align with previous studies, certain findings make this case rare. Based on previous research, low HCT levels, low HGB levels, hypocalcemia, and hypoglycemia are unexpected outcomes in this case report. Therefore, these findings make this case relatively specific owing to the uncommon findings in the blood tests. However, the patient’s low RBC, HCT, and HGB levels might be due to the patient's significantly low iron level (16 mcg/dL), which may have caused the anemia.

Studies indicate that a high level of HCQ is capable of causing false positives in buprenorphine and oxycodone [23]. Some serotonin–norepinephrine reuptake inhibitors (SNRIs) are reported to cause false positives in urine tramadol toxicology tests [24]. The patient was also under medication with prednisolone, multiple painkillers, and fluoxetine, and each of these drugs is capable of interfering with the urine drug test [25]. Therefore, findings in urine toxicology analysis are not as reliable as we could conclude. She also stated that she had taken naproxen recently for pain management, which can induce the false positive for benzodiazepines, but there is no evidence that a normal dose of these medications can cause the electrolyte imbalance in the patient. Since high levels of HCQ and azathioprine are capable of altering these tests and the patient indicates no history of overdose with drugs other than these two, the probability of these outcomes being related to other drugs is rare.

The complexity of the case owing to the intervention of multiple factors and lack of studies in this field resulted in difficulty justifying some of the characteristics of the patients, and therefore, further studies need to be accomplished in this field. These findings have the potential to prepare clinicians for similar situations that might occur.

Some limitations to our study should be taken into account. Owing to the lack of time and equipment in this case, we did not measure the serum level of azathioprine and HCQ in this patient. Additionally, the exact mechanism of hypocalcemia is not clear; it might be due to the azathioprine, HCQ, or other mentioned medications that the patient has been using.

Given the importance of and frequent usage of these two medications and the fact that physicians often prescribe HCQ and azathioprine together for autoimmune disease treatment, further investigation is needed to find the drug–drug interaction with these two drugs and their possible effect on each other and the human system.

## Limitations

Since this was an overdose case report, no prior laboratory tests were available before the incident to determine that electrocyte imbalance was purely an effect of overdose, but the last laboratory test, which was 80 days before the incident, was available and mentioned. The other thing that should be taken into account is the fact that despite the positive results in some urine toxicology tests, the concentration of these substances has not been measured in the blood, and based on the patient’s statement that she did not use any other medications other than HCQ and azathioprine to overdose, we consider the results might be owing to the false positive interactions as the effect of drug interactions. Owing to the retrospective nature of this case study, we acknowledge the constraints in data completeness, particularly in drug levels and interactions.

## Conclusion

Hydroxychloroquine and azathioprine toxicity are considered rare, but it is probable to increase in frequency given the prevalence and increase in autoimmune diseases and the increasing usage of these drugs in treating such diseases. In this case of overdose, hypokalemia, hypocalcemia, loss of consciousness, and hypotension were the main presentation of the patient, which has been managed successfully. Clinicians need to consider the unique effects of hydroxychloroquine and azathioprine poisoning. Although HCQ toxicity can be fatal owing to the imbalance of electrolytes and ions, this case was discharged with no major complications. In the case of azathioprine, the complications of overdose by the medication were not fatal. This case underscores the intricate interplay of polypharmacy in clinical outcomes, necessitating a deeper inquiry into drug–drug interactions.

Further research is essential to elucidate the mechanisms by which drug interactions contribute to electrolyte imbalances and other clinical findings in overdose cases. The differential diagnosis in overdose situations should be broadened to encompass potential interactions among prescribed medications, over-the-counter drugs, and substances not disclosed by patients. This case underscores the broader clinical imperative for healthcare providers to be alert to the risks of drug overdoses in chronic conditions and to consider atypical complications, such as hypocalcemia, in their differential diagnoses.

## Data Availability

The datasets are available from the corresponding author upon formal and logical request.
